# Interactions between mitochondrial dysfunction and other hallmarks of aging: Paving a path toward interventions that promote healthy old age

**DOI:** 10.1111/acel.13942

**Published:** 2023-07-27

**Authors:** Yuan Li, Laura Berliocchi, Zhiquan Li, Lene Juel Rasmussen

**Affiliations:** ^1^ Department of Cellular and Molecular Medicine, Center for Healthy Aging University of Copenhagen Copenhagen Denmark; ^2^ Department of Health Sciences University Magna Græcia of Catanzaro Catanzaro Italy

**Keywords:** ageing, aging, hallmarks of aging, mitochondria

## Abstract

Current research on human aging has largely been guided by the milestone paper “hallmarks of aging,” which were first proposed in the seminal 2013 paper by Lopez‐Otin et al. Most studies have focused on one aging hallmark at a time, asking whether the underlying molecular perturbations are sufficient to drive the aging process and its associated phenotypes. More recently, researchers have begun to investigate whether aging phenotypes are driven by concurrent perturbations in molecular pathways linked to not one but to multiple hallmarks of aging and whether they present different patterns in organs and systems over time. Indeed, preliminary results suggest that more complex interactions between aging hallmarks must be considered and addressed, if we are to develop interventions that successfully promote healthy aging and/or delay aging‐associated dysfunction and diseases. Here, we summarize some of the latest work and views on the interplay between hallmarks of aging, with a specific focus on mitochondrial dysfunction. Indeed, this represents a significant example of the complex crosstalk between hallmarks of aging and of the effects that an intervention targeted to a specific hallmark may have on the others. A better knowledge of these interconnections, of their cause‐effect relationships, of their spatial and temporal sequence, will be very beneficial for the whole aging research field and for the identification of effective interventions in promoting healthy old age.

## Graphical abstract

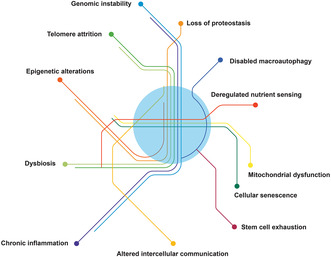

**Abbreviations**
AD: Alzheimer's disease
AKI: acute kidney injury
CKD: chronic kidney disease
DdCBE: derived cytosine base editor
DddA: double‐stranded DNA deaminase
FOX: forkhead box
HSCs: hematopoietic stem cells
IR/IGF1R: insulin/insulin‐like growth factor‐1 receptors
MAMs: mitochondria‐associated membranes
MDVs: mitochondrial‐derived vesicles
mitoARCUS: mitochondrial‐targeted meganucleases
mtDNA: mitochondrial DNA
NAFLD: non‐alcoholic fatty liver disease
NR: nicotinamide riboside
PD: Parkinson's disease
proteostasis: proteins homeostasis
ROS: reactive oxygen species
SASP: senescence‐associated secretory phenotype
TFAM: mitochondrial transcription factor A
TLS: translesion DNA synthesis
UPRmt: unfolded protein response

## 1 INTRODUCTION

**FIGURE 1:**
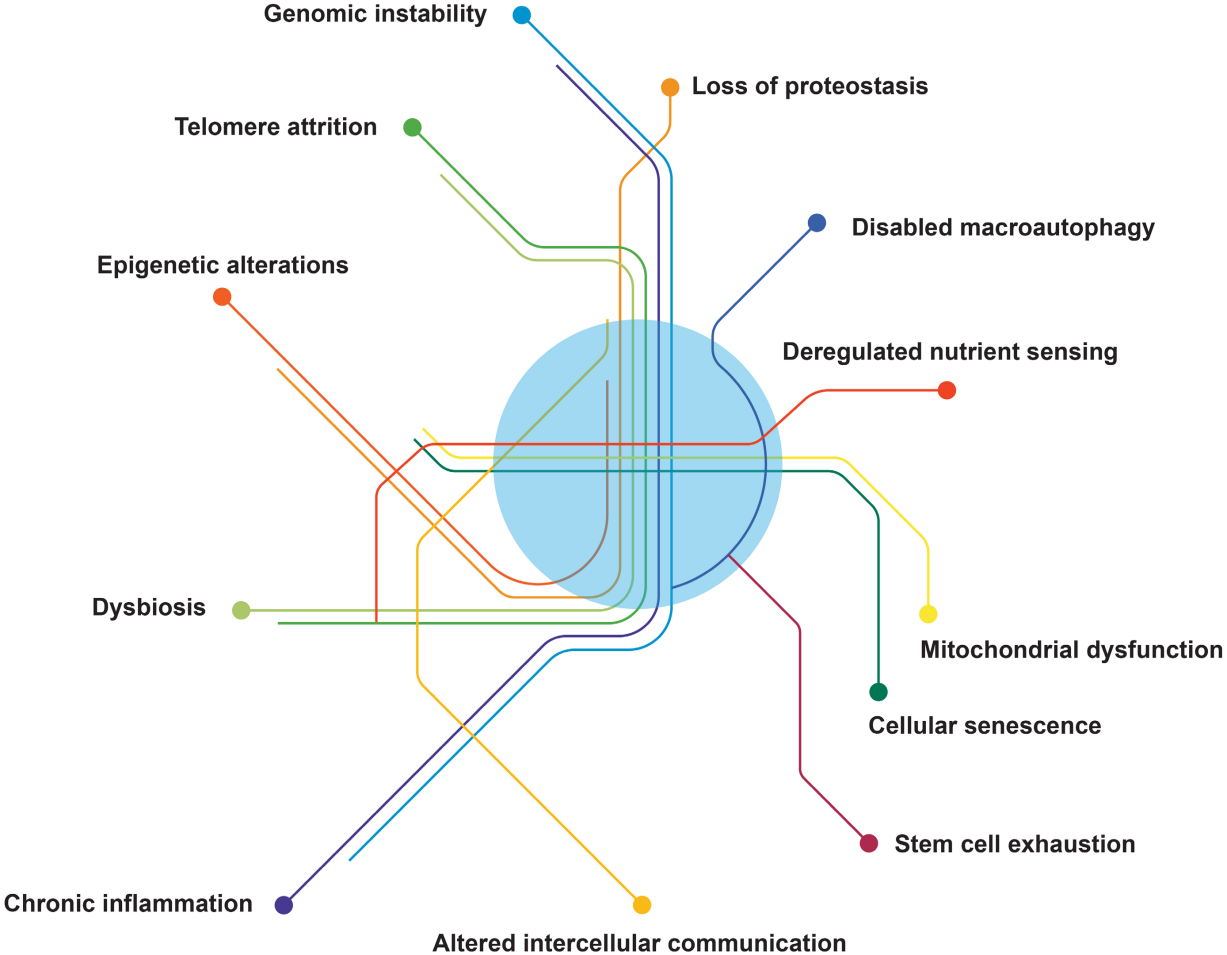
Complex etiology of human aging phenotypes. A complex interplay between the hallmarks of aging underlies the different trajectories of aging. Mitochondrial dysfunction represents a significant example of the interconnection between the different hallmarks.

Human aging is associated with increased risk and prevalence of chronic diseases, including dementia, coronary artery disease, atherosclerosis, diabetes, and several other common metabolic diseases. It is generally accepted that the high prevalence of these age‐related conditions reflects the time‐dependent accumulation of defects in specific cellular pathways and that the perturbations in these pathways cause aging‐associated phenotypes and diseases. A seminal paper published in 2013 by López‐Otín and his colleagues (Lopez‐Otin et al., 2013) identified nine aging‐related phenotypes and their associated molecular pathways as “the hallmarks of aging”: genomic instability, telomere attrition, epigenetic alterations, loss of proteostasis, deregulated nutrient‐sensing, mitochondrial dysfunction, cellular senescence, stem cell exhaustion, and altered intercellular communication (Lopez‐Otin et al., 2013). A more recent paper also by López‐Otín et al. proposed expanding the list of hallmarks to 12 to further include disabled macroautophagy, chronic inflammation, and dysbiosis in addition to the original nine (Lopez‐Otin et al., 2013, 2023; Figure 1). While these hallmarks have been extensively studied as independent determinants of aging, whether and how the aging hallmarks interact with and influence each other's impact on aging trajectories has received much less attention to date.

We underline strongly that the complex phenotypes of aging reflect equally complex underlying etiologies. As such, each distinct molecular pathway, but also the crosstalk and interplay mechanisms between them and their impact on individual aging trajectories, must be thoroughly understood if we hope to develop effective interventions against aging‐related diseases.

## 2 MITOCHONDRIAL DYSFUNCTION INTERACTS WITH OTHER HALLMARKS OF AGING

An abundance of evidence supports the idea that the etiology of aging trajectories is complex, multifactorial, and caused by perturbations in multiple interacting molecular pathways. For example, mitochondrial dysfunction, metabolic stress, and genomic instability are frequent concomitant biological features among old individuals, suggesting strong interactions between the pathways and determinants linked to these phenotypes.

**FIGURE 2:**
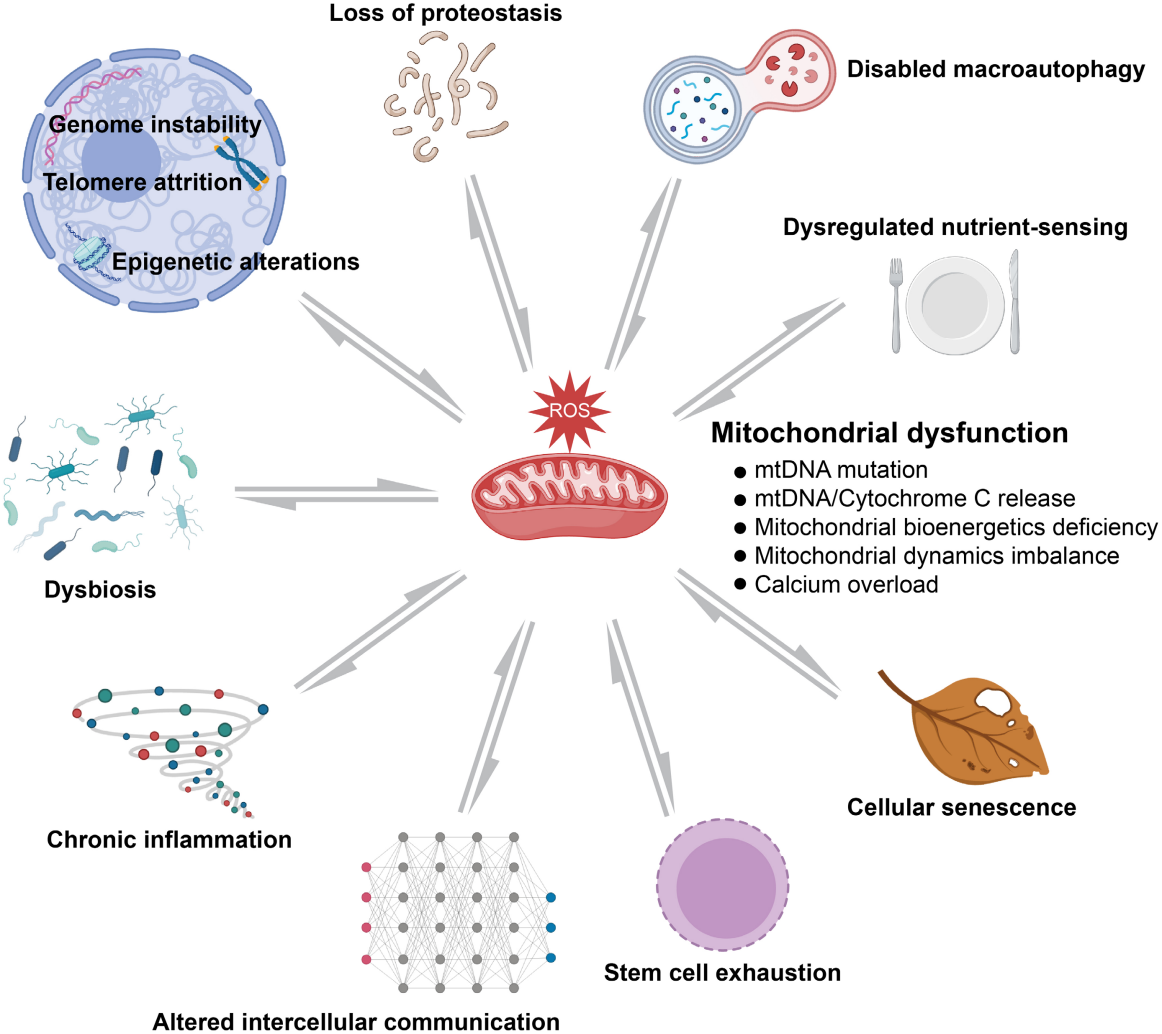
Bidirectional cross talk between mitochondrial dysfunction and hallmarks of aging. Mitochondrial dysfunction represents a significative example of the complex interaction between hallmarks of aging and shows that an intervention targeted to a specific hallmark may have significant effects on the others. Created with BioRender.com.

As one of the most important drivers of cellular aging, mitochondrial dysfunction is known to affect and interact with other aging hallmarks as well. By comprehensively understanding the underlying mechanisms through which mitochondrial dysfunction contributes to other hallmarks of aging, new potential antiaging strategies targeting mitochondrial dysfunction could be developed. Here, we will discuss how mitochondrial dysfunction interacts with other cellular drivers of aging (Figure 2).

Genomic instability, which refers to the accumulation of DNA damage and mutations, is one important primary driver of aging (Li et al., 2023), and several studies seem to connect this phenomenon to mitochondrial dysfunction. Studies in *Saccharomyces cerevisiae* have demonstrated that mitochondrial dysfunction induced by mitochondrial DNA (mtDNA) mutations downregulates iron‐sulfur cluster biogenesis, which in turn contributes to nuclear genome instability (Veatch et al., 2009). In the same model, mitochondrial dysfunction induced by either antimycin A or mtDNA depletion has been shown to be mutagenic and able to increase the frequencies of nuclear mutations (Rasmussen et al., 2003). At the molecular level, mitochondrial dysfunction has been linked to increased reactive oxygen species (ROS) and oxidative damage, activation of the error‐prone translesion DNA synthesis (TLS) pathway, decreased capacity to repair oxidative DNA damage, and nucleotide pool imbalances (Desler et al., 2007; Rasmussen et al., 2003). However, much evidence supports the idea that mitochondrial function and mtDNA stability are strongly linked to nuclear genome stability in a mutual way. In fact, unrepaired nuclear DNA damage can dysregulate critical metabolic pathways and induce mitochondrial dysfunction. Also, persistent nuclear DNA damage can inhibit TLS, inactivate or downregulate the TLS polymerase Rev1, decrease mitochondrial function, and induce metabolic stress (Fakouri et al., 2017; Martin‐Pardillos et al., 2017). These metabolic changes can be partly rescued by treatment with the NAD^+^ precursor nicotinamide riboside through a partial normalization of autophagy/mitophagy defects and mitochondrial dysfunction (Anugula et al., 2023). Further studies show that the crosstalk between nuclear DNA damage and mitochondrial homeostasis is mediated by the NAD^+^–SIRT1–PGC1a axis (Fang et al., 2016), suggesting that targeting this axis could be an effective strategy for reversing mitochondrial dysfunction associated with aging. The NAD‐dependent deacetylase SIRT1 acts through multiple downstream effectors and is known to induce degradation of the transcription factor HIF1a and deacetylation of the forkhead box (FOX) family transcription factors FOXO1 and FOXO3A (Canto et al., 2009; Fang et al., 2016; Gomes et al., 2013; Mouchiroud et al., 2013), which are critical master regulators of genes involved in aging, life span, and the response to oxidative stress (Kops et al., 2002; Nemoto & Finkel, 2002). Moreover, FOXO proteins have been shown to play a role in dietary restriction to modulate the expression of insulin/insulin‐like growth factor‐1 receptors (IR/IGF1R), AMPK, mTOR, JNK, as well as their downstream targets (Greer et al., 2007; Hay et al., 2011; Jiang et al., 2019; Kenyon et al., 2010; Mukhopadhyay et al., 2006; Robida‐Stubbs et al., 2012). The activation of FOXO signaling has been associated with extended life span and improved health span in model organisms, suggesting their crucial role in healthy aging. Dysregulation of FOXO‐regulated genes and pathways is widely implicated in age‐related diseases such as cancer, Alzheimer's disease (AD), and type 2 diabetesmellitus (Dansen et al., 2008; Du et al., 2021; Hwang et al., 2018; Manolopoulos et al., 2010). Mitochondrial dysfunction can also lead to other types of genomic instability, such as telomere loss and chromosome fusion (Liu et al., 2002). For instance, mitochondria can directly contribute to telomere attrition via ROS production (van der Rijt et al., 2020). However, telomere shortening can also affect mitochondria activity in multiple ways, such as the PGC‐1a/b and p53 pathways (Zhu et al., 2019). Mitochondria‐derived ROS can also impact cellular senescence by initiating or reinforcing the DNA damage response (DDR), thus resulting in a permanent growth arrest. In a study by Correia‐Melo et al. (2016), Passos and colleagues elucidated the involvement of mitochondria in an autoregulatory loop encompassing the DDR, mTOR‐PGC1β activation, and the generation of ROS. This loop leads to the sustained activation of DDR and plays a stabilizing role in cellular senescence. Importantly, targeted elimination of mitochondria from senescent cells proved effective in reversing numerous characteristics associated with the senescent phenotype in mice (Fielder et al., 2022). Moreover, mitochondrial dysfunction can trigger cellular senescence by increased mtDNA damage, alterations in energy metabolism, mitochondrial unfolded protein response (UPRmt)‐mediated releasing of IL‐6, and impaired mitophagy (Miwa et al., 2022). While mitochondrial dysfunction can drive cellular senescence, senescent cells themselves can further exacerbate mitochondrial dysfunction by releasing reactive molecules and factors such as senescence‐associated secretory phenotype (SASP) components (Fielder et al., 2022). This reinforcing loop between mitochondrial dysfunction and cellular senescence can contribute to the aging process and age‐related pathologies.

Also, epigenetic processes such as DNA methylation, histone modifications, and noncoding RNA expression are affected by mitochondrial dysfunction, thereby influencing gene expression and potentially impacting various cellular processes associated with aging and disease, as well as affecting mitochondrial health itself (D'Aquila et al., 2019; van der Rijt et al., 2020). However, the interactions between mitochondrial dysfunction and epigenetic alterations and, most important, their relevance for aging and age‐related diseases are still unclear, and therefore require more investigation.

Very close is the interaction between mitochondria and proteins homeostasis (proteostasis) and mitochondrial dysfunction can disrupt the balance between protein synthesis and the removal of damaged or misfolded proteins via multiple mechanisms, including energy depletion, oxidative stress, calcium dyshomeostasis, impaired mitophagy, and disrupted signaling pathways. The cross talk between mitochondria and proteostasis has been extensively studied in different models (Moehle et al., 2019), but a better understanding of the casualty of this connection is crucial to develop effective therapeutic tools for age‐related diseases, including neurodegenerative disorders.

The interconnection between mitochondria and the cellular quality control machinery is important for organelles such as mitochondria; thus, macroautophagy is a critical pathway for removing damaged, dysfunctional, or excess mitochondria (mitophagy). Mitochondrial dysfunction and impaired macroautophagy are interconnected processes that can mutually influence each other. Dysfunctional mitochondria can hinder the efficiency of macroautophagy, leading to the accumulation of damaged cellular components and accelerating the aging process (Rambold & Lippincott‐Schwartz, 2011; Roca‐Agujetas et al., 2019). Conversely, impaired macroautophagy can contribute to the accumulation of dysfunctional mitochondria, exacerbating mitochondrial dysfunction, and its associated detrimental effects. While defective mitophagy is clearly associated with various diseases (Chan, 2020; Cota et al., 2022; Doblado et al., 2021), clear evidence directing functional mitophagy to increased longevity is limited. Understanding the cause‐effect relationship between mitochondrial dysfunction and macroautophagy is crucial for developing therapeutic strategies targeting these processes to mitigate age‐related diseases and promote healthy aging. However, more research is needed to unravel the precise mechanisms and identify potential interventions for restoring or enhancing macroautophagy and mitochondrial health, resulting in aging benefits.

As individuals age, the depletion of stem cells becomes more prominent, leading to diminished regenerative potential (Shyh‐Chang et al., 2013). Stem cell exhaustion can be induced by mitochondrial dysfunction through several different mechanisms including metabolism changes, increased oxidative stress induced by mitochondrial ROS, accumulation of DNA damage, and impaired autophagy. The disordered mitochondrial dynamics and declined mitochondrial functions can induce the deterioration of adult stem cells as for instancein hematopoietic stem cells (HSCs). Specifically, when HSCs mitochondria become damaged with age, this will lead to a decline in their regenerative capacity, called HSCs aging, determining an elevated susceptibility to hematologic cancer in older people (Jaiswal et al., 2014). Moreover, elevated ROS levels in aged HSCs and neural stem cells (NSCs) in mice result in aberrant cell proliferation, increased risk of malignancy, and impaired self‐renewal ability of the stem cells (Sahin & Depinho, 2010). Moreover, aged HSCs display an accumulation of mtDNA mutations with an altered metabolism characterized by myeloid‐biased differentiation (Morganti & Ito, 2021). On the contrary, improved mitochondrial function has been found to enhance the functionality of stem cells and promote tissue regeneration in mammals. A notable example is the positive impact of short‐term caloric restriction on skeletal muscle stem cells in both young and aged mice. This beneficial effect is believed to stem from an increase in mitochondrial content and the promotion of oxidative metabolism (Bakhtiari et al., 2016). Similarly, in *Drosophila* intestinal stem cells, replenishing NAD^+^ levels enhance mitochondrial function, delays the onset of intestinal aging, and extends life span (Gomes et al., 2013). The interplay between mitochondrial dysfunction and stem cell exhaustion is complex, and the precise mechanisms underlying this relationship are still being investigated. Nonetheless, evidence suggests that maintaining mitochondrial health is crucial for preserving the regenerative capacity of stem cells and promoting healthy aging.

The aging process is also characterized by progressive alterations in intercellular communication systems (Lopez‐Otin et al., 2023). Mitochondrial dysfunction can alter intercellular communication by affecting various signaling mechanisms, including ROS, mtDNA, mitochondrial‐derived vesicles (MDVs) release, and metabolite signaling (McGuire, 2019; Picca et al., 2020; Pinti et al., 2014). Mitochondrial‐derived vesicles can induce the release of mtDNA fragments, metabolites, and proteins into the extracellular environment, which activates immune signaling pathways, disrupt the secretion of various molecules, and impair the transfer of functional mitochondria between cells (Dong et al., 2023; Picca et al., 2020). These disruptions can compromise cellular functions, impair tissue homeostasis, and possibly contribute to the development of various age‐related diseases such as AD and Parkinson's disease (PD). Understanding the impact of mitochondrial dysfunction on intercellular communication is crucial for comprehending the pathophysiology of these conditions and developing strategies to mitigate their effects. Interestingly, mitochondrial transfer can restore mitochondrial function in recipient cells and has therapeutic impact on different disease models for instance Pakinson's disease, stroke, and ischemia (Dong et al., 2023). Mitochondria injection by medial forebrain bundle injection can attenuate oxidative damage and the degeneration of dopaminergic neurons and improve locomotion (Chang et al., 2016). However, further research is needed to elucidate the underlying mechanisms and explore potential therapeutic interventions to restore proper intercellular communication in the context of mitochondrial dysfunction.

Mitochondrial dysfunction not only can affect the communication between cells, but also can dysregulate the response to nutrients and their energy balance. Dysregulated nutrient‐sensing is associated with aging and metabolic disorders, such as obesity, insulin resistance, and type 2 diabetes (Lopez‐Otin et al., 2023) and can be induced by mitochondrial dysfunction via impaired energy metabolism, increased reliance on glucose metabolism (glycolysis) rather than fatty acid oxidation, production of ROS, release of altered levels of metabolites and inflammation and stress responses. Mitochondria play vital roles as nutrient sensors (Ahn et al., 2008), and mitochondrial dysfunction has been extensively linked to metabolic diseases such as obesity (Bach et al., 2003). Mitochondria and endoplasmic reticulum interact at specific sites called mitochondria‐associated membranes (MAMs), facilitating the exchange of metabolites and calcium, and emerging evidence indicate MAMs as central hubs for hepatic insulin signaling and nutrient sensing processes (Theurey & Rieusset, 2017). Moreover, the main nutrient sensing pathways (insulin/IGF1, mTOR, AMPK) are also tightly connected to mitochondrial function and mitophagy and efforts to preserve mitochondrial function, reduce oxidative stress, and restore metabolic flexibility hold promise for mitigating dysregulated nutrient‐sensing and providing therapeutics approaches for the associated metabolic disorders (Andreux et al., 2013).

Several recent studies highlight multiple gut microbiome trajectories of aging, depending on microbial communities in the body, particularly within the gut microbiota, whose imbalance may lead to dysbiosis (Lopez‐Otin et al., 2023). Mitochondrial dysfunction is commonly observed in the aging gastrointestinal tract (Houghton et al., 2018), but the gut‐brain axis is emerging as a complex bidirectional pathway, linking the gut microbiota with the brain and also implicating mitochondrial function (Barber et al., 2021). The two best‐known neurodegenerative diseases, AD and PD, are accompanied by both mitochondrial dysfunction and poor microbiota (Kramer, 2021). Emerging evidence suggests that mitochondrial dysfunction can contribute to dysbiosis and alter the composition and function of the gut microbiota through various mechanisms, including altered host–microbiota crosstalk, metabolic changes, oxidative stress, intestinal barrier dysfunction, and immune dysregulation. Disturbances in microbial communities can have implications for overall health and contribute to the development of various diseases and conditions (Kramer, 2021). Also, the interplay between mitochondrial dysfunction and dysbiosis is complex and bidirectional: While mitochondrial dysfunction can contribute to dysbiosis, dysbiosis itself can also affect mitochondrial function by producing metabolites and microbial‐derived factors (Barber et al., 2021).

Chronic low‐grade inflammation (inflammaging) is a hallmark feature of the aging process (Franceschi & Campisi, 2014). Mitochondrial dysfunction can play a significant role in chronic inflammation by affecting different pathways, including increased ROS production, inflammasome activation, the imbalance between mitophagy and mitochondrial biogenesis, activation of nuclear factor‐kappa B (NF‐κB) pathway, and dysregulated metabolism (Marchi et al., 2023). Of note, the translocation of mtDNA into cytosol can increase the level of pro‐inflammatory cytokines (Miller et al., 2021). A T cell‐specific defect in the mitochondrial transcription factor A (TFAM) can induce an aging phenotype, including increased circulating cytokines in mice (Desdin‐Mico et al., 2020). Strategies targeting mitochondrial quality control, reducing oxidative stress, and modulating inflammation‐associated signaling pathways can help attenuate chronic inflammation and alleviate the associated pathological conditions. However, it is important to note that mitochondrial dysfunction is often interconnected with other factors contributing to chronic inflammation, such as immune dysregulation and environmental factors (i.e., diet and dysbiosis). Therefore, a more comprehensive understanding of the complex interactions between mitochondrial dysfunction and chronic inflammation is necessary to develop effective therapeutic interventions.

## 3 THE ORGAN‐SPECIFIC PROFILE OF MITOCHONDRIAL DYSFUNCTION AND HALLMARKS OF AGING

The interplay between mitochondrial dysfunction and other hallmarks of aging highlights the complex and interconnected nature of the aging process. Understanding these reciprocal relationships is crucial for developing interventions that target multiple hallmarks, to mitigate age‐related decline and promote healthy aging. Moreover, very recent work has highlighted how distinct organs and systems age differently, identifying organ/tissue‐specific aging clocks (Nie et al., 2022), which may predict chronic disease and mortality (Tian et al., 2023). This provides the first evidence for an even more complex scenario where the classical hallmarks of aging might have distinct relevance depending on the organ or system. Initial work in mice has also started to investigate the organ‐specific temporal signatures of the hallmarks of aging (Schaum et al., 2020), and it is becoming clear the importance of understanding the temporal sequence of these interactions (Rando & Wyss‐Coray, 2021). This understanding will be essential to develop more personalized and effective therapies, toward one or more specific organ/system at a specific time.

From this perspective, it will be important to understand also how the different hallmarks of aging interact and impact the aging process of different tissues, organs, and systems. Briefly, we will highlight here some of the evidence on the role of mitochondrial dysfunction in various organs and tissues, focusing on those more relevant in aging‐related disorders such as neurodegenerative, cardiovascular, metabolic, musculoskeletal, and immune system diseases. As the cell powerhouse, mitochondria generate energy in the form of ATP via oxidative phosphorylation, and this function has a strong impact on specific organs. For instance, the brain, which requires a significant amount of energy to function properly, is very dependent on mitochondria but also particularly vulnerable to oxidative damage. Mitochondrial dysfunction has been widely found in normal brain aging, brain injury, and neurological disorders, including AD, PD, and Huntington's disease (Grimm & Eckert, 2017; Paradies et al., 2011). Mitochondrial‐targeted strategies such as mitophagy induction can reduce AD pathologies and show cognitive benefits in AD mice models (Fang et al., 2019; Hayakawa et al., 2016). Moreover, neurons can release damaged mitochondria and transfer them to astrocytes for disposal and recycling (Davis et al., 2014), as well as astrocytes may release extracellular mitochondrial particles that enter neurons to support cell viability and recovery after stroke (Hayakawa et al., 2016). Mitochondria represent a promising therapeutic target in brain aging and age‐related neurodegenerative disorders (Cunnane et al., 2020; Fairley et al., 2022). The heart is also an organ that requires a lot of energy to pump blood and maintain cardiovascular function. In fact, mitochondrial dysfunction can lead to heart failure, myocardial infarction, and several cardiovascular diseases including cardiomyopathies and arrhythmias (Bisaccia et al., 2021; Peoples et al., 2019). Mitochondrial abnormalities underlining these conditions include impaired mitochondrial electron transport chain activity, increased formation of ROS, shifted metabolic substrate utilization, aberrant mitochondrial dynamics, and altered ion homeostasis. Therefore, mitochondrial dysfunction seems to be an important target for therapy to improve cardiac function directly (Brown et al., 2017; Rudiger et al., 2011). However, further studies are still necessary to investigate whether restoration of mitochondrial function could provide potential new therapeutic modalities against heart failure.

In the liver, an essential organ for several metabolic functions and rich in mitochondria, age‐related diseases include nonalcoholic fatty liver disease (NAFLD), alcoholic liver disorders, hepatitis, fibrosis, and cirrhosis. Some of these, including liver cirrhosis and liver failure, can be due to mitochondrial dysfunction and have been associated with increased mitochondrial ROS production and impaired oxidative phosphorylation (Middleton & Vergis, 2021). Several studies have shown beneficial effects of mitochondrially targeted antioxidants (e.g., MitoQ10) in preclinical models through the reduction of transaminitis, hepatic steatosis, liver peroxidation, and inflammation (Hao et al., 2018; Hoyt et al., 2017).

Like the other metabolically active organs, also the pancreas undergoes changes with aging in volume, structure, and perfusion, which can lead to insufficient functionality. In the case of the endocrine pancreas, an age‐related decline in the proliferative potential of insulin‐producing β‐cells is well‐documented and was proposed to contribute to the increased prevalence of type 2 diabetes in the older people (Helman et al., 2016). The molecular basis for the age‐related decline of β‐cell proliferation is still incompletely understood but mitochondrial dysfunction has been shown to impair insulin secretion and lead to glucose dysregulation, contributing to the development of diabetes (Nile et al., 2014). Moreover, several studies suggest that partial mtDNA depletion observed in aging has a direct and detrimental effect on pancreatic beta cell function, glucose‐stimulated insulin secretion, and decreased ATP content (Gauthier et al., 2009; Ihm et al., 2006).

Aging‐associated alterations also occur in the kidney, leading to decreased glomerular filtration rate, tubular dysfunction, and glomerulosclerosis. Furthermore, kidney aging has important implications for aging‐associated comorbidities, especially cardiovascular diseases and diabetes (Lee et al., 2019). In the kidney, mitochondrial dysfunction can result in electrolyte imbalances, fluid overload, and other complications leading to renal failure and playing a critical role in the progression of acute kidney injury (AKI) and chronic kidney disease (CKD) (Bhatia et al., 2020). Several mitochondria‐targeted antioxidants have been explored as potential therapies in experimental models of AKI and CKD (Chacko et al., 2010; Dare et al., 2015). Although mitochondria‐focused pharmacological approaches in kidney disease have been investigated only recently, several clinical trials are ongoing to investigate their short‐ and long‐term effects (Tanriover et al., 2023).

Progressive loss of muscle mass and physical function (e.g., sarcopenia) is one of the distinctive features of aging and may lead to important mobility impairment. This seems to be paralleled by a progressive loss of mitochondrial function (i.e., mitochondrial respiratory activity; Short et al., 2005). Mitochondrial dysfunction can lead to muscle weakness, fatigue, and other muscular disorders but an increase in physical activity, achieved through regular exercise, can largely improve mitochondrial function and negate the decline of muscle function in older people (Grevendonk et al., 2021), therefore, leaving still unclear the causative relationship between these changes. In fact, old adults are generally more inactive with advancing age, which in turn decreases functional fitness and mitochondrial functions. However, preclinical and clinical data suggest that aging *per se* is associated with muscular mitochondrial dysfunction, reduced mitochondrial genes expression, insufficient autophagy/mitophagy, and reduced NAD^+^ levels (Dao et al., 2020). Therefore, although increasing physical activity through regular training partially protects against the age‐related declines in mitochondrial and muscle health, mitochondria represent a promising therapeutic target to counteract the age‐related decline in skeletal muscle health and to preserve physical function and performance.

Activation of the innate immune system occurs with aging, resulting in a low‐grade chronic pro‐inflammatory state (inflammaging), which is one of the 12 hallmarks of aging. Although the exact causal role of this condition on health outcomes is still unclear, most age‐related diseases (diabetes, cancer, metabolic syndromes, AD, and heart disease) share an inflammatory component. The exact relationship between mitochondria and immune function in aging still needs deep investigation, but several data suggest that mitochondrial dysfunction within aging can influence immune cell function and contribute to immune system dysregulation (McGuire, 2019). Defective mitochondria in immune cells can impair their ability to mount an effective immune response, leading to increased susceptibility to infections, impaired wound healing, and chronic inflammation. Mitochondrial dysfunction also drives premature aging (Peter, 2021). The release of mtDNA, ROS, ATP, and mitochondrial proteins in the cytosol, as well as the release of mitochondria or their fragments in tissue/bloodstream, may act as potent immunomodulators (Caicedo et al., 2021; Morganti & Ito, 2021). Targeting mitochondrial dysfunction and pathways appears to be a valuable strategy to modulate the immune system dysregulation associated with the aging process (Yao et al., 2018).

## 4 MITOCHONDRIA‐TARGETED INTERVENTIONS

**FIGURE 3:**
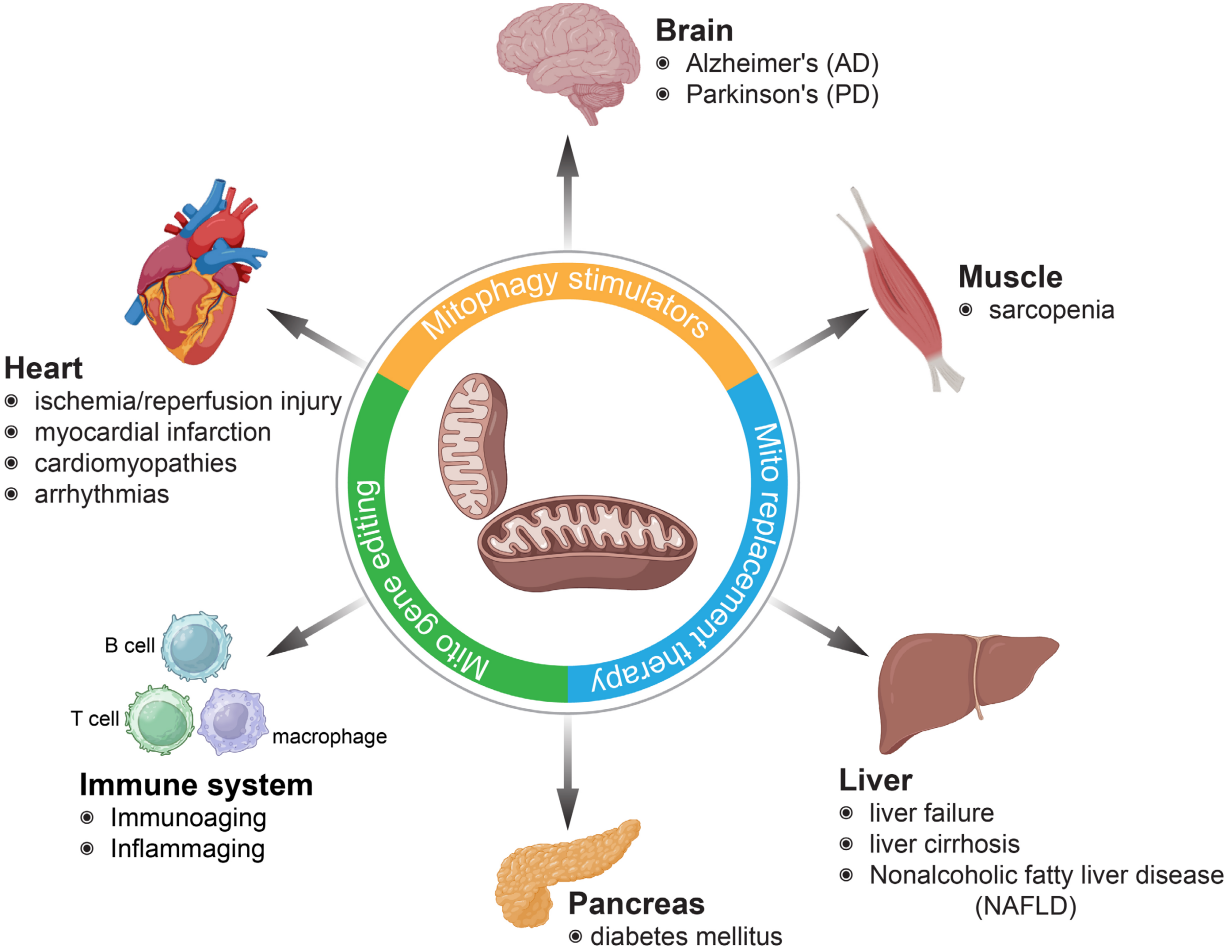
Examples of the role of mitochondrial dysfunction in different organ aging and aging‐related diseases. Distinct organs and systems age differently, and the classical “hallmarks of aging” might have distinct relevance depending on the organ or system.

Mitochondria‐targeted interventions have emerged as a promising area of research in aging and age‐related diseases. Several strategies have been explored to specifically deliver therapeutic agents or compounds to mitochondria, aiming to enhance their function, reduce oxidative stress, and restore cellular homeostasis. These interventions can encompass a range of approaches, including small molecules, peptides, antioxidants, and gene therapy (Figure 3). Mitophagy stimulators have, in recent years, been proposed as a promising strategy to attenuate aging and neurodegenerative diseases, such as AD. NAD^+^ precursors, such as nicotinamide riboside, nicotinamide mononucleotide, and analogs, have been widely investigated in aging and neurodegenerative disease models (Bertoldo et al., 2020; Fang et al., 2019). It shows many benefits for mitochondria and rescues the compromised diversity and perturbated microbial compositions in AD (Chu et al., 2022). Another promising small molecule is Urolithin A that showed dramatic power in stimulating mitophagy, increasing muscle functions, and mitigating amyloid‐β and tau pathology (Ryu et al., 2016). Interestingly, peptides for improving mitochondrial functions highlight the synthetic tetrapeptide, elamipretide (SS‐31), which improves mitochondrial functions by interacting with the phospholipid cardiolipin enriched on the inner mitochondrial membrane (Chavez et al., 2020). The mitochondria replacement therapy has also been implicated as a potential path toward healthy mitochondria in inherited or age‐related mitochondrial dysfunction/diseases (Herbert & Turnbull, 2018). Another route would be the genome editing for mitochondrial genes, for example, in several mouse models, using mitochondrially targeted zinc‐finger nucleases (Gammage et al., 2018), mitochondrial‐targeted meganucleases (mitoARCUS) (Zekonyte et al., 2021), double‐stranded DNA deaminase (DddA)‐derived cytosine base editor (DdCBE) (Silva‐Pinheiro et al., 2022) or an adenine base editor (ABE8e), and a potent AAV9 delivery of RNA‐guided Cas9 nuclease (Reichart et al., 2023). This has been primarily used to correct severe inherited genetic diseases (Kang et al., 2016), but the rapid development of new editing tools might enable the thorough investigations of age‐related mitochondrial perturbations in preclinical in vivo studies and provide enough evidence for future applications in humans.

Mitochondria‐targeted interventions offer a compelling avenue for combating aging and age‐related diseases by directly addressing the underlying mitochondrial impairments. While many of these approaches are still in the early stages of research and development, they hold great potential for improving mitochondrial health, preserving cellular function, and extending a healthy life span. Continued advancements in understanding mitochondrial biology and refining targeted intervention strategies may lead to transformative therapies that have a profound impact on human health and aging.

## 5 CONCLUSION AND FUTURE PERSPECTIVES

Altogether, we highlighted the interactions between mitochondrial dysfunction and other hallmarks of aging, as well as their impact on different organs and tissues. Given the ubiquitous critical role of mitochondria in cellular energy production and homeostasis, mitochondrial dysfunction can have far‐reaching consequences throughout the body, affecting various physiological processes and contributing to the development of numerous diseases. Understanding and addressing mitochondrial dysfunction is therefore crucial for maintaining organ health and overall healthy aging.

A deep understanding of these complex inter‐relationships between the hallmarks of aging will be critical, as we strive to develop interventions that reduce or delay morbidity and increase quality of life in old age (Keshavarz et al., 2023; Longo et al., 2015). Two of the most promising drugs currently tested for intervention, rapamycin and metformin, confirm the tight interconnection between the different hallmarks of aging. They are both examples of repurposed drugs, which by acting on key targets in distinct tissues and organs, can exert beneficial effects on life span and/or health span. By inhibiting mTORC1, a central and ubiquitous cellular sensor finely modulated by multiple inputs and regulating multiple downstream targets and pathways, rapamycin has been shown to extend life span and improve health span in several preclinical models by ameliorating multiple hallmarks of aging, such as altered nutrient sensing, stem cell dysfunction, cellular senescence, impaired intercellular communication, impaired proteostasis and autophagy, and mitochondrial dysfunction (Partridge et al., 2020). Based on preclinical studies, rapamycin and its analogs appear to have beneficial effects in many different organs and systems including brain, heart, intestine, liver, skeletal muscle, senescent skin cells, and the immune system. However, the approved use of rapamycin and rapalogs for transplantation and cancer treatment highlighted relevant side effects. Chronic treatment of humans with high doses of rapalogs is associated with deleterious metabolic consequences including hyperlipidemia, hypercholesterolemia, hypertriglyceridemia, insulin resistance, and glucose intolerance, which seem to be reduced by intermittent or low‐dose regimens of the drugs (Mannick & Lamming, 2023). Metformin, a first‐line antidiabetic drug, has been shown to increase life span (although not in all preclinical models) and reduce the incidence of aging‐related diseases by interacting with several hallmarks of aging. Through AMPK activation, it has been suggested to protect against oxidative stress, preserve mitochondrial function, reduce telomere shortening, inhibit cellular senescence prevent inflammation, and shift the gut microbiota composition (Campisi et al., 2019; Partridge et al., 2020). However, also metformin presents several side effects, some of which may have severe consequences and a better understanding of the mechanisms underlying its beneficial effects on aging is required. Most probably, the best strategy for aging intervention will be the optimal combination of synergistic treatments for the specific individual phenotype.

The aging field is ripe for new developments and mechanistic insights. We anticipate that additional hallmarks of aging are likely to be identified, as research on human aging continues to progress. However, as the complexity of aging is becoming more and more clear, new multidimensional analytical frameworks (Keshavarz et al., 2023) and possibly new approaches to the hallmarks of aging and their re‐organization (Gems & de Magalhaes, 2021) will be needed to progress in the understanding of this process and in the advancement of effective intervention strategies.

In closing, critical questions about aging research and the search for effective interventions need to be addressed, including: (i) What are the challenges and roadblocks that are delaying or undermining the development of effective interventions for aging‐related morbidities? (ii) Are the current hallmarks good enough to catch the complexity of aging and move forward our understanding of this process? (iii) Are cell type‐, tissue‐ and organ‐specific processes important determinants of aging trajectories? and (iv) If they are, how do these processes interact between them and impact the organism aging? Answers to these and other important queries will move us toward a more comprehensive understanding of human aging and treatment of aging‐associated diseases.

## AUTHOR CONTRIBUTIONS

All authors contributed extensively to writing, editing, and approving the final version.

## CONFLICT OF INTEREST STATEMENT

The authors state that the research was carried out without any affiliations or financial associations that could be considered as a possible conflict of interest.

## Data Availability

Data sharing is not applicable to this article as no new data were created or analyzed in this study.
